# The position of patients for safe central venous catheterization via the internal jugular vein: prospective observational study of critically ill patients

**DOI:** 10.1186/2036-7902-7-S1-A15

**Published:** 2015-03-09

**Authors:** Young Soon Cho, Jong Bin Lee

**Affiliations:** 1 Department of Emergency Medicine, Soonchunhyaung University Bucheon Hosptial, Bucheon, Korea

## Background

Carotid artery injuries are common complications during catheterization of the internal jugular vein.

## Objective

The purpose of this study is to determine the best position for the reduction of carotid artery injuries. Unlike a previous study, only critically ill patients who needed central venous catheterization in an emergency medical center were included.

## Patients and methods

Eight positions were tested in each patient. The positions were classified by maneuver and ultrasound images of each position were stored. Two factors were determined at each position: “safety width” (the part of the internal jugular vein that did not overlap with the carotid artery) and “overlap width” (the part of the internal jugular vein that did overlap with the carotid artery).

## Results

Compared with the neutral bed position, safety widths were significantly larger in the Trendelenburg position, and there were no statistical difference in overlap widths. Compared with the non-head rotation position, safety widths were smaller and overlap widths were significantly larger in the 45 head rotation positions. Safety widths did not statistically change safter adjustments for ultrasound probe level. However changing the ultrasound probe level from the base of Sedillot’s triangle to thyroid cartilage significantly decreased overlap widths. Overall, The group of Trendeleberg position, with non-head rotation, whose ultrasound probe level was thyroid cartilage had largest safety widths among 8 groups.

## Conclusion

Among the eight positions tested, the Trendelenburg position (with limited head rotation and adjustments for ultrasound probe level) can reduce carotid artery injuries and increase the successful catheterization of the jugular vein in critically ill patients.

